# Diabetes knowledge and attitudes among pharmacy students in Northern Cyprus: a cross-sectional study

**DOI:** 10.1186/s12909-026-10145-6

**Published:** 2026-08-12

**Authors:** Ahmed Alaqab, Mohammed Alfaqeeh, Anas Hamad, Niveen M. E. Abu-Rmeileh, Aysel Pehlivanlı, Nevzat Birand, Meryem Deniz Aydın

**Affiliations:** 1 Department of Pharmacy, Faculty of Medical Sciences, Al-Ruwad University, Taiz, Republic of Yemen; 2 Department of Pharmacology and Clinical Pharmacy, Faculty of Pharmacy, Universitas Padjadjaran, Bandung, Indonesia; 3 Drug Supply Department, Hamad Medical Corporation, Doha, Qatar; 4 College of Pharmacy, QU Health, Qatar University, Doha, Qatar; 5 Department of Public Health, College of Health Sciences, QU Health, Qatar University, Doha, Qatar; 6 Department of Pharmacology, Faculty of Pharmacy, Başkent University, Ankara, Turkey; 7 Clinical Pharmacy and Drug Information Center, Ankara Hospital, Baskent University, Ankara, Turkey; 8 Department of Clinical Pharmacy, Faculty of Pharmacy, Near East University, Nicosia, North Cyprus Turkey

**Keywords:** Diabetes mellitus, Public health, Health education, Pharmacy students

## Abstract

**Background:**

Diabetes mellitus (DM) is a major global public health challenge associated with substantial morbidity, mortality, and healthcare costs. As pharmacists play a key role in diabetes management, assessing pharmacy students’ knowledge and attitudes is essential to ensure effective future care. This study aimed to evaluate student pharmacists’ knowledge and attitudes toward DM in Northern Cyprus.

**Methods:**

A cross-sectional study was conducted between November 2023 and January 2024 among fourth- and fifth-year pharmacy students from three universities in Northern Cyprus. Diabetes-related knowledge was assessed using a structured questionnaire, and attitudes were evaluated using the Diabetes Attitude Scale (DAS). Knowledge scores were calculated across four domains, and participants’ responses were compared according to academic year and sociodemographic characteristics using Chi-square tests, independent-samples t tests, and one-way analysis of variance (ANOVA). Effect sizes and post-hoc analyses were performed where applicable.

**Results:**

A total of 265 students participated. Overall, student pharmacists demonstrated a moderate level of diabetes-related knowledge (19.20/26), with stronger performance in risk factors and signs and symptoms and lower performance in general knowledge and selected complications. Knowledge was highest for risk factors (5.81/7) and signs and symptoms (5.78/7), followed by complications (4.30/6), while general knowledge showed the lowest score (3.50/6). Students demonstrated strong awareness of common symptoms and risk factors, although gaps remained in treatment-related knowledge and selected diabetes complications. Fifth-year students showed significantly better awareness of retinopathy and neuropathy compared with fourth-year students. The mean DAS scores indicated generally positive attitudes, with the highest score observed for the need for special training (4.53 ± 0.62) and the lowest for the value of tight glycemic control (3.15 ± 0.64). Significant differences were observed across DAS subscales (*p* < 0.001).

**Conclusions:**

Student pharmacists demonstrated generally good diabetes-related knowledge and positive attitudes; however, important gaps remained in practical aspects of diabetes management and selected complications. Addressing these gaps through experiential and patient-centered learning approaches may further enhance students’ preparedness to deliver effective diabetes care.

**Supplementary Information:**

The online version contains supplementary material available at https://doi.org/10.1186/s12909-026-10145-6.

## Introduction

Diabetes mellitus (DM) remains a major global public health concern, requiring long-term management, patient education, and continuous monitoring to prevent complications and improve quality of life [1, 2]. The complexity of diabetes care has increased significantly in recent years, placing greater demands on healthcare systems and professionals [3]. In this context, effective disease management extends beyond pharmacological treatment to include patient counseling, lifestyle modification, and ongoing follow-up, highlighting the need for well-prepared healthcare providers who can deliver comprehensive and patient-centered care [4, 5]. The burden of diabetes is particularly substantial in the region. According to the International Diabetes Federation (IDF), Türkiye has one of the highest diabetes prevalence rates in Europe, with approximately one in seven adults affected by the disease [6]. As the prevalence of diabetes continues to rise, healthcare systems face increasing challenges related to disease prevention, treatment, and long-term management, further emphasizing the need for competent healthcare professionals who are equipped to provide effective diabetes care [7].

In response to these growing healthcare demands, pharmacists are increasingly recognized for their expanding role in the management of chronic diseases such as diabetes [8]. In addition to medication dispensing, pharmacists contribute to medication therapy management, patient education, monitoring of therapeutic outcomes, and supporting patients in self-management practices [9–11]. Their accessibility within healthcare systems positions them as key contributors to improving treatment adherence, glycemic control, and overall patient outcomes [12]. As a result, the role of pharmacists has evolved from a product-oriented function to a more patient-centered clinical role, emphasizing the importance of communication skills, clinical knowledge, and professional attitudes in delivering effective care [13].

Given these evolving responsibilities, pharmacy education plays a critical role in preparing future pharmacists to meet the demands of modern healthcare systems [14]. It is essential that pharmacy students develop not only a strong foundation of theoretical knowledge but also the clinical competencies and attitudes necessary for managing chronic conditions such as diabetes [15]. In particular, knowledge and attitudes are closely linked to professional performance, influencing decision-making, patient interaction, and the quality of care provided [16]. Educational strategies have increasingly shifted toward more interactive and experiential approaches, including simulation-based learning, case-based discussions, and interprofessional education, all of which aim to enhance students’ clinical skills, confidence, and readiness for practice [17, 18].

Despite these advancements in pharmacy education, previous studies have reported variability in students’ knowledge and attitudes toward diabetes, with persistent gaps in key areas such as insulin use, complication awareness, and dietary management [19, 20]. These findings suggest that current educational approaches may not fully address all aspects required for effective diabetes care [21]. Furthermore, much of the available evidence has been generated from relatively homogeneous educational settings, with limited exploration of contexts involving diverse and multicultural student populations [22, 23]. The importance of strengthening diabetes-related competencies is further underscored by evidence from the Eastern Mediterranean region, where diabetes continues to impose a substantial burden on patient outcomes and healthcare systems [24, 25]. Additionally, a study from the region have suggested that educational environment and year of study may influence students’ learning experiences, wellbeing, and professional development [26].This is an important consideration, as differences in educational background and cultural perspectives may influence students’ understanding, attitudes, and preparedness for clinical practice.

Northern Cyprus represents a distinctive educational setting, with universities offering internationally oriented pharmacy programs that attract students from diverse backgrounds [27]. Differences in curricula, teaching methods, clinical training opportunities, and language of instruction may influence students’ learning experiences and preparedness for practice [28]. Assessing pharmacy students’ knowledge and attitudes toward DM is therefore important to evaluate the effectiveness of current educational approaches. Comparing fourth- and fifth-year students may also provide insight into the impact of educational progression and clinical exposure. However, evidence remains limited regarding whether pharmacy students in Northern Cyprus, an international and multicultural higher-education setting, possess sufficient applied diabetes knowledge and patient-centered attitudes before graduation. Given the limited evidence from Northern Cyprus, this study aimed to assess diabetes-related knowledge and attitudes among fourth- and fifth-year pharmacy students in Northern Cyprus and to identify educational gaps that may inform curriculum improvement.

## Methods

### Study design and setting

This study employed a cross-sectional design to assess the knowledge and attitudes of pharmacy students toward DM, and was reported in accordance with the Strengthening the Reporting of Observational Studies in Epidemiology (STROBE) guidelines. The study was conducted over a three-month period, from November 2023 to January 2024, across three universities in Northern Cyprus: the European University of Lefke, Near East University, and Cyprus International University. These institutions offer accredited pharmacy programs and enrol a diverse population of local and international students. The study targeted students in the clinical phase of their training, where coursework and practical exposure to chronic disease management, including DM, are emphasized.

### Study population and sampling

The study population consisted of fourth- and fifth-year pharmacy students enrolled at the selected universities during the study period. The sample size was determined using Raosoft^®^ online software [29], assuming a 5% margin of error, 95% confidence level, 50% response distribution, and a total population size of 708 students, resulting in a minimum required sample size of 250 participants. A convenience sampling approach was employed, and all eligible students were invited to participate. Inclusion criteria were limited to students registered in the fourth or fifth year of the pharmacy program. Students enrolled in the first to third years of the pharmacy program, students from universities not included in the study, students who declined to provide informed consent, and questionnaires with incomplete responses were excluded from the analysis. Participation was voluntary, and all responses were collected anonymously.

### Study instruments and data collection

Data were collected using both paper-based questionnaires and an online Google Form. The questionnaires were available in English and Turkish, and participants were allowed to complete the questionnaire in their preferred language. Paper questionnaires were administered in person during class sessions at some universities, while students at other universities received the online survey link. For the online questionnaire, all survey items were configured as mandatory fields, and participants could not proceed to the next section or submit the questionnaire without completing all required items. For paper-based questionnaires, responses were reviewed for completeness at the time of collection, and participants were asked to complete any unintentionally omitted items before submission whenever possible. Consequently, only fully completed questionnaires were included in the final dataset, and no missing data were present in the analyzed sample. The full versions of the questionnaires in both English and Turkish can be found in Supplementary File 1.

The first questionnaire was designed to assess students’ knowledge of DM and was developed based on previously published studies to reflect the educational objectives and clinical context of pharmacy students in Northern Cyprus [30–32]. The questionnaire was not adopted from a single validated instrument; rather, relevant items addressing general knowledge, risk factors, signs and symptoms, and complications of diabetes were selected and refined by the research team. Content validity was established through review by an expert panel of three clinical pharmacists, and face validity was further evaluated by two independent clinical pharmacists. The individual content validity index (I-CVI) was calculated according to Lynn’s criteria [33]. Following minor revisions based on expert feedback, all items achieved an I-CVI of 1.0, indicating complete agreement among experts regarding item relevance and clarity. The questionnaire was translated into Turkish using a forward–backward translation method to ensure linguistic accuracy, and the Turkish version was pilot-tested among a small group of pharmacy students to assess clarity and comprehension before the main study. The pilot test included 15 pharmacy students who were not included in the final study sample, and their responses were excluded from all subsequent analyses. The pilot testing was conducted to assess questionnaire clarity and comprehension, whereas the item–total correlation analysis was subsequently performed using data from the main study sample. The knowledge assessment tool consisted of two main sections: the first section collected sociodemographic information, including gender, age, body mass index (BMI), marital status, academic year, grade point average (GPA), smoking status, alcohol use, and familiarity with diabetes, while the second section comprised 26 items assessing knowledge of DM, divided into general knowledge (6 items), risk factors (7 items), signs and symptoms (7 items), and complications (6 items). Responses were formatted as multiple-choice or true/false questions, with one correct answer per item.

Students’ attitudes toward DM were assessed using the validated Turkish version of the Diabetes Attitude Scale-3 (DAS-3) [34]. The DAS consists of 33 items distributed across seven subscales, including need for special training, patient compliance, seriousness of type 2 diabetes, blood glucose control and complications, psychosocial impact of diabetes, patient autonomy, and team care. Each item was rated on a 5-point Likert scale ranging from 1 (strongly disagree) to 5 (strongly agree) [35]. Scoring and interpretation were performed according to the validated Turkish DAS-3 methodology.

### Ethical approval

This study was conducted in accordance with the principles of the Declaration of Helsinki and received ethical approval from the Institutional Review Board (IRB) of Near East University (Approval No: NEU/2023/119–1791). Prior to participation, all students were provided with information about the study, including its objectives, voluntary nature, and assurances of anonymity and confidentiality. Written informed consent was obtained from all participants before data collection.

### Data analysis

Construct validity of the knowledge questionnaire was assessed using item–total correlation analysis in the main study sample, with Pearson’s correlation coefficients calculated between each item and the total score of its respective domain. Items with correlation coefficients greater than 0.30 were considered acceptable and retained in the final questionnaire. Internal consistency reliability was evaluated using Cronbach’s alpha, with values ≥ 0.60 indicating acceptable reliability [36]. Knowledge scores were calculated by assigning one point for each correct response and zero for incorrect or “don’t know” answers, as both were considered to indicate the absence of demonstrated correct knowledge for the purpose of the total knowledge score. This resulting in a possible total score ranging from 0 to 26, with higher scores indicating better knowledge. Knowledge levels were categorized as good, moderate, or poor. A score of 21–26 (≥ 80%) was considered good knowledge, 16–20 (60–79%) was considered moderate knowledge, and 0–15 (< 60%) was considered poor knowledge [37]. Attitudes toward DM were assessed using the DAS, and mean scores were calculated for each participant and for each subscale.

Descriptive statistics were used to summarize participants’ sociodemographic characteristics, knowledge scores, and attitude responses. Frequencies and percentages were reported for categorical variables, while means and standard deviations were used for continuous variables.

The Chi-square test was applied to examine associations between categorical variables and to compare knowledge of diabetes-related factors between fourth- and fifth-year students. Independent samples t-tests were used to compare mean attitude scores between the two academic years. Prior to conducting parametric analyses, assumptions of normality and homogeneity of variance were assessed. Normality was evaluated using visual inspection of histograms and Q–Q plots together with the Shapiro–Wilk test, while homogeneity of variance was assessed using Levene’s test. The assumptions were considered adequately satisfied before applying independent-samples t-tests and one-way ANOVA. When ANOVA results were statistically significant, Tukey’s honestly significant difference (HSD) test was performed for post-hoc pairwise comparisons. One-way analysis of variance (ANOVA) and Chi-square tests were further used to assess associations between DAS scores and sociodemographic characteristics. Effect sizes were calculated to quantify the magnitude of observed differences. Cohen’s d was reported for independent-samples t-tests and eta-squared (η²) for one-way ANOVA. Cohen’s d values of 0.20, 0.50, and 0.80 were interpreted as small, medium, and large effects, respectively, while η² values of 0.01, 0.06, and 0.14 were interpreted as small, medium, and large effects. Test statistics and degrees of freedom were reported together with p-values for all t-tests and ANOVA results. A p-value of < 0.05 was considered statistically significant. Given the exploratory nature of the analyses, no formal adjustment for multiple comparisons was applied; therefore, statistically significant findings should be interpreted cautiously in view of the potential for increased Type I error. All statistical analyses were performed using STATA Statistical Software for Data Science version 17 (StataCorp LLC, College Station, TX, USA) [38].

## Results

### Validity and reliability assessment

The knowledge questionnaire demonstrated acceptable construct validity, with item–total correlation coefficients ranging from 0.34 to 0.64 across all 26 items (Supplementary File 2 (Table S1)). All items exceeded the predefined validity threshold and were retained in the final instrument. Content validity was excellent, with all questionnaire items achieving an I-CVI of 1.0. The overall internal consistency of the knowledge questionnaire was acceptable (Cronbach’s α = 0.74). Detailed reliability coefficients for the knowledge questionnaire and DAS subscales are presented in Supplementary File 2 (Table S2).

### Participant characteristics

A total of 265 pharmacy students participated in the study, including 35.1% fourth-year and 64.9% fifth-year students. Participants were recruited from three universities in Northern Cyprus, with the majority enrolled at Near East University. Most participants were female (57.0%), and the majority were aged between 20 and 25 years (77.4%). A smaller proportion were aged above 25 years (20.7%), while very few were under 20 years (1.9%). In terms of academic performance, GPA distribution varied across study years, with a higher proportion of fifth-year students reporting GPAs between 2.5 and 3.0, while a notable proportion of fourth-year students had GPAs above 3.0. Most participants had a normal BMI (64.2%), with approximately one-fifth classified as overweight and a smaller proportion as obese. Regarding lifestyle characteristics, smoking and alcohol use were reported by 29.9% and 26.4% of participants, respectively. Additionally, more than two-thirds of students (68.3%) reported knowing someone diagnosed with DM. Detailed sociodemographic characteristics are presented in Table 1.

**Table 1:** Demographic characteristics of student pharmacists

Variable	4th year *n* (%)	5th year *n* (%)	Total *n* (%)	*p*-value
Gender				
Female	56 (60.2)	95 (55.2)	151 (57.0)	0.439
Male	37 (39.8)	77 (44.8)	114 (43.0)	
Age (years)				
< 20	1 (1.1)	4 (2.3)	5 (1.9)	0.080
20–25	79 (84.9)	126 (73.3)	205 (77.4)	
> 25	13 (14.0)	42 (24.4)	55 (20.7)	
Marital status				
Single	69 (74.2)	122 (70.9)	191 (72.0)	0.179
Married	9 (9.7)	7 (4.1)	16 (6.1)	
In a relationship	15 (16.1)	43 (25.0)	58 (21.9)	
GPA				
< 2.0	9 (9.7)	8 (4.7)	17 (6.4)	0.312
2.0–2.5	23 (24.7)	44 (25.6)	67 (25.3)	
2.5–3.0	25 (26.9)	70 (40.7)	95 (35.8)	
> 3.0	36 (38.7)	50 (29.1)	86 (32.5)	
BMI (kg/m²)				
< 18.5	11 (11.8)	13 (7.6)	24 (9.1)	0.684
18.5–24.9	52 (55.9)	115 (66.9)	167 (63.0)	
25–29.9	26 (28.0)	32 (18.6)	58 (21.9)	
≥ 30	4 (4.3)	12 (7.0)	16 (6.3)	
Smoking				
Yes	24 (25.8)	53 (30.8)	77 (29.9)	0.418
No	69 (74.2)	119 (69.2)	188 (70.1)	
Alcohol use				
Yes	28 (30.1)	42 (24.4)	70 (26.4)	0.339
No	65 (69.9)	130 (75.6)	195 (73.6)	
Know someone with DM				
Yes	63 (67.7)	118 (68.6)	181 (68.3)	0.873
No	30 (32.3)	54 (31.4)	84 (31.7)	

*Abbreviations: BMI *body mass index, *GPA *grade point average, *DM *diabetes mellitus

### Knowledge of diabetes mellitus

Detailed responses and comparisons between academic years are presented in Table 2. Overall, student pharmacists achieved a knowledge score of 19.20/26 (73.8%). Domain-level analysis showed the highest performance in risk factors (5.81/7; 83.0%) and signs and symptoms (5.78/7; 82.5%), whereas general knowledge demonstrated the lowest score (3.50/6; 58.4%). Detailed domain-level knowledge scores are presented in Supplementary File 2 (Table S3), while item-level scoring results are presented in Supplementary File 2 (Table S4).

**Table 2:** Knowledge of diabetes mellitus among student pharmacists

Variable	4th year *n* (%)	5th year *n* (%)	*P*-value
General Knowledge			
Is Type 1 DM Insulin-dependent?			
Yes*	78 (83.9)	140 (81.4)	0.510
No	10 (10.7)	16 (9.3)	
Don’t know	5 (5.4)	16 (9.3)	
Is Type 2 DM Insulin-resistant?			
Yes*	73 (78.5)	131 (76.2)	0.906
No	12 (12.9)	24 (13.9)	
Don’t know	8 (8.6)	17 (9.9)	
Does only Type 1 DM present with ketonuria?			
Yes	32 (34.4)	62 (36.1)	0.431
No*	35 (37.6)	52 (30.2)	
Don’t know	26 (28.0)	58 (33.7)	
Are autoantibodies present in Type 1 DM?			
Yes*	57 (61.3)	98 (57.0)	< 0.001
No	28 (30.1)	14 (8.1)	
Don’t know	8 (8.6)	60 (34.9)	
Insulin administration routes			
Subcutaneous (SC)*	60 (64.5)	122 (70.9)	0.475
Intramuscular (IM)	24 (25.8)	53 (30.8)	0.475
Intravenous (IV)	37 (39.8)	47 (27.3)	0.052
Diet recommendations			
35% carbohydrate + 50% fat + 15% protein	14 (15.1)	29 (16.9)	0.837
15% carbohydrate + 35% fat + 50% protein	56 (60.2)	83 (48.3)	0.083
50% carbohydrate + 35% fat + 15% protein*	23 (24.7)	60 (34.9)	0.118
Risk Factors			
Is age > 45 years a risk factor?			
Yes*	76 (81.7)	153 (88.9)	0.088
No	12 (12.9)	9 (5.2)	
Don’t know	5 (5.4)	10 (5.8)	
Is obesity (BMI ≥ 30 kg/m²a risk factor?			
Yes*	81 (87.1)	149 (86.6)	0.705
No	7 (7.5)	10 (5.8)	
Don’t know	5 (5.4)	13 (7.6)	
Is positive family history a risk factor?			
Yes*	88 (94.6)	145 (84.3)	0.040
No	2 (2.2)	16 (9.3)	
Don’t know	3 (3.2)	11 (6.4)	
Is gestational diabetes a risk factor?			
Yes*	70 (75.3)	128 (74.4)	0.763
No	12 (12.9)	27 (15.7)	
Don’t know	11 (11.8)	17 (9.9)	
Is hypertension a risk factor?			
Yes*	67 (72.0)	136 (79.1)	0.368
No	17 (18.3)	21 (12.2)	
Don’t know	9 (9.7)	15 (8.7)	
Is carbohydrate-rich diet a risk factor?			
Yes*	75 (80.6)	136 (79.1)	0.937
No	11 (9.7)	21 (12.2)	
Don’t know	7 (7.5)	15 (8.7)	
Is lack of physical activity a risk factor?			
Yes*	82 (88.2)	154 (89.5)	0.817
No	6 (6.5)	8 (4.7)	
Don’t know	5 (5.4)	10 (5.8)	

*P*-values were derived from Pearson's chi-square tests comparing the distribution of responses across all response categories between fourth-year and fifth-year students

*Abbreviations: DM *diabetes mellitus, *BMI *body mass index, *SC *subcutaneous, *IM *intramuscular, *IV *intravenous

*P*-values <0.05 indicate statistical significance

*, Correct answers

A high proportion of participants correctly identified that type 1 DM is insulin-dependent (82.3%) and that type 2 DM is associated with insulin resistance (77.0%). In addition, most students recognized age greater than 45 years (86.4%) and obesity (86.8%) as important risk factors. No statistically significant differences were observed between fourth- and fifth-year students for most knowledge items. However, significant differences were identified for knowledge of autoantibody presence in type 1 diabetes (*p* = 0.0001) and positive family history as a risk factor (*p* = 0.0403), with slightly higher correct responses among fourth-year students. Regarding treatment-related knowledge, subcutaneous injection was the most frequently identified route of insulin administration among both groups. In contrast, knowledge of diet recommendations showed variability among students, indicating some uncertainty in this area.

Knowledge regarding diabetes signs, symptoms, and complications was generally high, particularly for common symptoms and microvascular complications. Nevertheless, lower awareness was observed for limb gangrene (66.4%) and ketoacidosis (73.6%), indicating persistent gaps in complication-related knowledge. Significant differences between academic years were observed for knowledge of retinopathy (*p* = 0.0201) and neuropathy (*p* = 0.0061), with higher awareness among fifth-year students. No statistically significant differences were found for most other signs, symptoms, and complications. Overall, the findings indicate adequate knowledge of core diabetes concepts, although several treatment- and complication-related topics warrant additional educational reinforcement. Detailed results are presented in Table 3.

**Table 3:** Knowledge of signs, symptoms, and complications of diabetes mellitus among student pharmacists

Variable	4th year *n* (%)	5th year *n* (%)	*P*-value
Polyuria			
Yes*	77 (82.8)	140 (81.4)	0.937
No	4 (4.3)	9 (5.2)	
Don’t know	12 (12.9)	23 (13.4)	
Polydipsia			
Yes*	86 (92.5)	152 (88.4)	0.288
No	3 (3.2)	14 (8.1)	
Don’t know	4 (4.3)	6 (3.5)	
Polyphagia			
Yes*	72 (77.4)	141 (82.0)	0.364
No	9 (9.7)	18 (10.5)	
Don’t know	12 (12.9)	13 (7.6)	
Dry mouth			
Yes*	80 (86.0)	144 (83.7)	0.470
No	4 (4.3)	14 (8.1)	
Don’t know	9 (9.7)	14 (8.1)	
Tiredness, fatigue, lethargy			
Yes*	83 (89.2)	142 (82.6)	0.203
No	5 (5.4)	21 (12.2)	
Don’t know	5 (5.4)	9 (5.2)	
Delayed wound healing			
Yes*	77 (88.8)	147 (85.5)	0.069
No	4 (4.3)	15 (8.7)	
Don’t know	12 (12.9)	10 (5.8)	
Frequent infections			
Yes*	66 (71.0)	124 (73.3)	0.674
No	8 (8.6)	19 (11.2)	
Don’t know	19 (20.4)	29 (15.5)	
Retinopathy			
Yes*	64 (68.8)	136 (79.1)	0.020
No	6 (6.5)	16 (9.3)	
Don’t know	23 (24.7)	20 (11.6)	
Nephropathy			
Yes*	73 (78.5)	126 (73.3)	0.072
No	7 (7.5)	24 (14.0)	
Don’t know	13 (14.0)	12 (7.0)	
Neuropathy			
Yes*	63 (67.7)	126 (73.3)	0.006
No	6 (6.5)	25 (14.3)	
Don’t know	24 (25.8)	21 (12.2)	
Ketoacidosis			
Yes*	70 (75.3)	125 (72.7)	0.124
No	5 (5.4)	22 (12.8)	
Don’t know	18 (19.4)	25 (14.5)	
Hypoglycemia			
Yes*	69 (74.2)	112 (70.3)	0.795
No	15 (16.1)	31 (18.0)	
Don’t know	9 (9.7)	20 (11.6)	
Hyperglycemia			
Yes*	77 (82.8)	137 (79.7)	0.103
No	5 (5.4)	22 (12.8)	
Don’t know	11 (11.8)	13 (7.6)	
Limb gangrene			
Yes*	58 (62.4)	118 (68.6)	0.080
No	9 (9.7)	25 (14.5)	
Don’t know	26 (28.0)	29 (16.9)	

*P*-values were derived from Pearson's chi-square tests comparing the distribution of responses across all response categories between fourth-year and fifth-year students

*P*-values <0.05 indicate statistical significance

*, Correct answers

### Attitudes toward diabetes mellitus

Student pharmacists demonstrated positive attitudes toward DM, as reflected by the DAS scores. The mean DAS score was comparable between fourth-year and fifth-year students, with no statistically significant difference observed(t(263) = 0.82, *p* = 0.412, Cohen’s d = 0.10). A statistically significant difference in DAS scores was found based on gender, with female students demonstrating higher mean scores compared to male students (t(263) = 2.81, *p* = 0.005, Cohen’s d = 0.35). In addition, significant differences were observed across GPA categories (F(3, 261) = 5.47, *p* = 0.002, η² = 0.059), with higher academic performance associated with more positive attitudes. The effect size for the gender comparison was small to moderate (Cohen’s d = 0.35), while the GPA comparison demonstrated a moderate effect size (η² = 0.059). Post-hoc analysis showed that students with GPA > 3.0 had significantly higher DAS scores than those with GPA < 2.0 (*p* = 0.001) and those with GPA between 2.0 and 2.5 (*p* = 0.009). Furthermore, students who reported knowing someone with DM had significantly higher DAS scores compared to those who did not (t(263) = 2.67, *p* = 0.008, Cohen’s d = 0.34). No statistically significant differences were observed for other variables, including age, marital status, BMI, smoking status, alcohol use, and exercise. Detailed comparisons of DAS scores according to sociodemographic and lifestyle characteristics, including test statistics, effect sizes, and post-hoc comparisons where applicable, are presented in Table 4.

**Table 4:** Comparison of Diabetes Attitude Scale (DAS) scores according to sociodemographic and lifestyle characteristics of student pharmacists

Variable	Mean ± SD	Test Statistic	*p*-value	Effect Size	Post-hoc
Academic Year					
4th (*n* = 93)	3.76 ± 0.38	t(263) = 0.82	0.412	d = 0.10	—
5th (*n* = 172)	3.73 ± 0.41				
Gender					
Female (*n* = 151)	3.81 ± 0.39	t(263) = 2.81	0.005*	d = 0.35	—
Male (*n* = 114)	3.67 ± 0.41				
GPA					
< 2.0 (*n* = 17)	3.47 ± 0.42	F(3,261) = 5.47	0.002*	η² = 0.059	3.0 vs. < 2.0 (*p* = 0.001) 3.0 vs. 2.0–2.5 (*p* = 0.009)
2.0–2.5 (*n* = 67)	3.68 ± 0.35				
2.5–3.0 (*n* = 95)	3.76 ± 0.41				
> 3.0 (*n* = 86)	3.84 ± 0.39				
Marital Status					
In a relation (*n* = 58)	3.73 ± 0.40	F(2,262) = 1.82	0.164	η² = 0.014	—
Married (*n* = 16)	3.92 ± 0.54				
Single (*n* = 191)	3.77 ± 0.32				
Age					
Under 20 (*n* = 5)	4.04 ± 0.30	F(2,262) = 2.40	0.093	η² = 0.018	—
20–25 (*n* = 205)	3.72 ± 0.39				
Above 25 (*n* = 55)	3.81 ± 0.42				
BMI					
< 18.5 (*n* = 24)	3.78 ± 0.28	F(3,261) = 1.82	0.143	η² = 0.021	—
18.5–24.9 (*n* = 166)	3.75 ± 0.38				
25–29.9 (*n* = 59)	3.77 ± 0.43				
≥ 30 (*n* = 16)	3.57 ± 0.52				
Smoking					
No (*n* = 188)	3.76 ± 0.42	t(263) = 0.49	0.626	d = 0.08	—
Yes (*n* = 77)	3.73 ± 0.34				
Alcohol					
No (*n* = 195)	3.73 ± 0.40	t(263) = 0.96	0.339	d = 0.15	—
Yes (*n* = 70)	3.79 ± 0.39				
Exercise					
No (*n* = 148)	3.77 ± 0.40	t(263) = 1.20	0.230	d = 0.14	—
Yes (*n* = 117)	3.72 ± 0.39				
Knowing Someone with Diabetes					
No (*n* = 84)	3.65 ± 0.42	t(263) = 2.67	0.008*	d = 0.34	—
Yes (*n* = 181)	3.79 ± 0.38				

Independent-samples t-tests were used for comparisons between two groups, whereas one-way ANOVA was used for comparisons involving more than two groups. Tukey's honestly significant difference (HSD) test was performed for post-hoc pairwise comparisons following significant ANOVA results. Cohen's d was reported for independent-samples t-tests and eta-squared (η²) for one-way ANOVA analyses

*Abbreviations: DAS *Diabetes Attitude Scale, *BMI *body mass index, *GPA *grade point average

*, Statistical significance was set at *p* < 0.05

### Diabetes Attitude Scale (DAS) subscale analysis

Analysis of the DAS subscales showed variability in mean scores across the seven domains. The highest mean score was observed for “need for special training” (4.53 ± 0.62), indicating strong agreement among students on the importance of specialized training in diabetes care. High scores were also observed for “team care” (4.42 ± 0.63). This was followed by “psychosocial impact of diabetes” (3.94 ± 0.57), “patient autonomy” (3.90 ± 0.71), and “patient compliance” (3.78 ± 0.71). In contrast, lower mean scores were observed for “seriousness of type 2 diabetes” (3.45 ± 0.66) and “value of tight glycemic control” (3.15 ± 0.64), suggesting comparatively lower levels of agreement in these areas. All DAS-3 subscale scores were significantly higher than the neutral midpoint value of 3.0 (all *p* ≤ 0.003), indicating generally positive attitudes toward diabetes care among student pharmacists. Detailed results of all DAS subscales and one-sample t-test results, including t-statistics, degrees of freedom, and p-values are presented in Table 5, while item-level responses are provided in Supplementary File 2 (Table S5).

**Table 5:** DAS-3 subscale scores and one-sample t-test results

Subscale	Mean ± SD	t-statistic	*P*-value
Need for Special Training	4.53 ± 0.62	19.24	< 0.001
Patient Compliance	3.78 ± 0.71	15.65	< 0.001
Seriousness of type 2 DM	3.45 ± 0.66	10.49	< 0.001
Value of Tight Control	3.15 ± 0.64	3.70	0.003
Psychosocial Impact of DM	3.94 ± 0.57	26.87	< 0.001
Patient Autonomy	3.90 ± 0.71	20.63	< 0.001
Overall DAS Score	3.74 ± 0.40	29.96	< 0.001

Separate one-sample t-tests were conducted for each DAS-3 subscale, comparing the observed mean score with the neutral midpoint value of 3.0

*Abbreviations: DAS *Diabetes Attitude Scale, *SD *standard deviation, *DM *diabetes mellitus

## Discussion

The present study evaluated the knowledge and attitudes of fourth- and fifth-year pharmacy students in Northern Cyprus toward DM. Overall, student pharmacists demonstrated generally good knowledge and positive attitudes toward diabetes care. Fifth-year students showed greater awareness of long-term diabetes complications, particularly retinopathy and neuropathy, compared with fourth-year students. Although these differences were modest, they may reflect the added benefit of further coursework and clinical exposure during later stages of training. High awareness of major complications such as nephropathy, retinopathy, and neuropathy suggests a solid foundation in core clinical concepts. However, some knowledge gaps remained, highlighting the need for targeted educational strategies to strengthen preparedness for diabetes management in practice.

In terms of knowledge, students demonstrated strong awareness of common symptoms and major complications of diabetes mellitus, including polydipsia, polyuria, nephropathy, retinopathy, and neuropathy. This suggests that core clinical concepts related to diabetes are well covered within existing educational programs. Moreover, fifth-year students generally demonstrated higher levels of knowledge than fourth-year students, particularly regarding long-term complications. This pattern likely reflects the impact of increased academic exposure and clinical training as student progress through the curriculum [39]. However, not all knowledge domains followed this pattern, and fourth-year students demonstrated comparable or slightly higher performance in some individual items. This may reflect more recent exposure to specific diabetes-related coursework, whereas fifth-year students may have shifted their focus toward broader clinical competencies during advanced training [40]. Furthermore, variations in course sequencing and curricular emphasis across participating universities may have influenced knowledge retention and performance in specific domains.

Nevertheless, certain areas of knowledge were less well understood, particularly those related to insulin administration routes and dietary management. These findings are consistent with previous studies reporting variability in pharmacy students’ understanding of practical aspects of diabetes care. For example, a study among fourth- and fifth-year pharmacy students in Türkiye reported a mean overall diabetes knowledge score of 34.07/40, with scores of 2.88/3 for diabetes risk factors and 2.70/3 for diabetes symptoms [41]. Although direct numerical comparison is limited by differences in questionnaire structure and scoring, the similarly strong performance in risk-factor and symptom-related knowledge suggests a consistent pattern across the two student populations. Compared with findings from other settings, including studies conducted among pharmacy students in Turkey and elsewhere, the level of knowledge observed in our study was somewhat lower in specific domains [42]. For example, the findings indicate that student pharmacists possessed a generally good level of diabetes-related knowledge, which is consistent with studies conducted in Eastern Mediterranean countries, where pharmacy and other health-related students demonstrated adequate overall knowledge of diabetes mellitus, although important gaps persisted in specific domains of disease management and complications [43, 44]. In the present study, knowledge was strongest for diabetes risk factors and signs and symptoms, whereas lower scores were observed for selected aspects of treatment-related knowledge and complications. These findings suggest that while core concepts of diabetes are well understood, additional emphasis on applied diabetes management may further strengthen students’ preparedness for future clinical practice. Such differences may be attributable to variations in educational approaches and experiential learning opportunities, particularly the timing and intensity of clinical training components, which can influence students’ ability to translate theoretical knowledge into practice [45].

The findings related to insulin administration further highlight the need for improved practical training. Although subcutaneous administration was widely recognized, awareness of other routes was more limited. Similar observations have been reported in previous studies, where gaps in procedural knowledge were attributed to insufficient hands-on training [46]. This underscores the importance of incorporating more experiential learning opportunities, such as simulation-based training and clinical case discussions, to enhance students’ practical competencies and confidence in diabetes management [47, 48].

Regarding attitudes, the study revealed generally positive perceptions toward diabetes care among student pharmacists. Students expressed strong agreement with principles of patient-centered care, teamwork, and the importance of effective communication. These findings align with existing literature suggesting that pharmacy students are increasingly oriented toward patient-centered practice models [49, 50]. Such attitudes are essential for effective chronic disease management, where patient engagement and interdisciplinary collaboration play critical roles [51]. The comparatively lower score for the “Value of Tight Glycaemic Control” subscale is noteworthy given its importance in diabetes management. This finding may reflect greater familiarity with individualized treatment approaches and highlights the need to reinforce evidence-based glycaemic management principles within pharmacy education [52]. Female students also demonstrated significantly higher DAS scores than male students. Although some differences were statistically significant, their relatively small effect sizes suggest that their practical or educational significance may be limited. Therefore, these findings should be interpreted cautiously and not assumed to represent meaningful differences in diabetes-related attitudes. This finding may also reflect differences in attitudes toward patient-centered care, empathy, or engagement with chronic disease management, which may be influenced by social and cultural factors [53]. However, these factors were not directly assessed in the present study and warrant further investigation.

Differences in attitudes between academic years were observed across several DAS subscales. Fifth-year students demonstrated higher scores in the ‘Need for Special Training’, ‘Seriousness of Type 2 Diabetes’, and ‘Psychosocial Impact of Diabetes’ subscales, whereas fourth-year students showed slightly higher scores in the ‘Patient Autonomy’ subscale. These findings may reflect differences in educational experiences and clinical exposure between academic years; however, the cross-sectional design of the study does not allow conclusions regarding causality or changes over time [54, 55]. Similar trends have been reported in studies examining the impact of clinical rotations on students’ professional development [56–58]. In addition, the bilingual survey format (English/Turkish) may have influenced how some items were interpreted despite the use of forward–backward translation, particularly in a multicultural student population with varying language preferences and proficiency levels. Although forward–backward translation was used, bilingual administration may have introduced subtle differences in item interpretation between the English and Turkish versions, potentially affecting response consistency. The multicultural composition of the student population may also have influenced the findings, as students from diverse national and educational backgrounds may differ in their prior exposure to diabetes education, healthcare systems, and cultural perspectives on chronic disease management [59].

This study has several limitations that should be considered when interpreting the findings. First, the study was conducted among students from only three universities in Northern Cyprus, with a relatively large proportion of participants from a single institution, which may limit the generalizability of the results. Another limitation is the use of convenience sampling, which may have introduced selection bias. Students who were available and willing to participate may have differed from non-participants in their interest in diabetes-related topics, academic engagement, or attitudes toward patient care. Consequently, the study sample may not be fully representative of all pharmacy students in Türkiye, and the findings should be interpreted with caution when generalizing to broader student populations. In addition, because the exact number of students invited to participate was not recorded, a response rate could not be reliably calculated. Therefore, the potential impact of non-response bias could not be formally assessed and should be considered when interpreting the findings. Moreover, the item–total correlation analysis used to assess the construct validity of the knowledge questionnaire was conducted using the main study sample rather than an independent validation sample; therefore, the psychometric findings should be interpreted cautiously and warrant confirmation in an independent sample. The use of bilingual (English/Turkish) questionnaires may also have introduced minor variations in question interpretation and response consistency. In addition, the use of a voluntary sampling approach may have introduced selection bias, as students with greater interest or confidence in diabetes-related topics may have been more likely to participate. The cross-sectional design captures data at a single point in time and does not allow for causal inferences or assessment of changes over time. Furthermore, the reliance on self-reported questionnaires may introduce response bias, with participants potentially overestimating their knowledge and attitudes. Although validated instruments were used, they may not fully reflect students’ actual clinical competencies. Moreover, scoring both incorrect and “don’t know” responses as zero did not distinguish between incorrect knowledge and uncertainty, which may have limited the interpretation of specific knowledge gaps. Additionally, the analyses conducted in this study were primarily exploratory and descriptive in nature, focusing on group comparisons rather than the identification of independent predictors of attitudes toward diabetes. Therefore, multivariable regression analyses were not performed to adjust for potential confounding variables such as gender, academic year, and GPA. Therefore, the observed associations should be interpreted with appropriate caution. Future studies using larger and more diverse samples should consider multivariable regression models to better evaluate the independent contribution of these factors while controlling for potential confounding. Additionally, multiple statistical comparisons were conducted, which may increase the risk of Type I error. As no formal adjustment for multiple testing was applied, the findings should be interpreted with appropriate caution. Future studies involving larger and more diverse samples may benefit from multivariable modeling to evaluate the independent contribution of sociodemographic and educational factors while controlling for potential confounding variables. Finally, clinical performance and communication skills were not directly assessed, which are essential components of pharmacy practice and diabetes care.

These findings have important implications for pharmacy education and public health. The observed gaps in knowledge related to insulin administration routes, dietary recommendations, and diabetes complications highlight the need for more targeted and practice-oriented educational interventions. Strengthening experiential learning components, including simulation-based training, case-based learning, and interprofessional education, may improve students’ competence in these areas and help bridge the gap between theoretical knowledge and clinical application [60]. Earlier and more consistent exposure to clinical scenarios may further support students’ preparedness for managing complex conditions such as diabetes [61]. From a public health perspective, pharmacists play a crucial role in diabetes prevention, patient education, and long-term disease management [62]; therefore, ensuring that pharmacy graduates are equipped with both strong clinical knowledge and appropriate patient-centered attitudes is essential for improving health outcomes and supporting healthcare systems. Educational approaches that emphasize communication skills, patient counseling, and collaborative practice should be further integrated into pharmacy curricula, particularly in the context of chronic disease management [63, 64]. Future efforts should focus on developing and evaluating innovative educational models that effectively combine theoretical instruction with practical training across different stages of pharmacy education [65], while expanding research to include larger and more diverse student populations and longitudinal designs to better understand how knowledge, attitudes, and clinical competencies evolve over time [66, 67]. Additionally, incorporating objective assessments of clinical performance alongside self-reported measures may provide a more comprehensive evaluation of educational outcomes and inform continuous curriculum improvement [68, 69].

## Conclusion

This study showed that pharmacy students in Northern Cyprus demonstrated generally good knowledge and positive attitudes toward diabetes mellitus. However, important gaps were identified in practical aspects of diabetes care, particularly insulin administration, dietary management, and awareness of certain complications. Fifth-year students demonstrated better knowledge in selected areas, suggesting that increased clinical exposure and educational progression may enhance preparedness for practice. These findings support strengthening pharmacy curricula through earlier clinical exposure, simulation-based training, case-based learning, and greater emphasis on patient counseling and chronic disease management. Future curriculum development in Northern Cyprus should incorporate these educational strategies to better prepare pharmacy graduates for their expanding role in diabetes prevention, patient education, and long-term disease management.

## Supplementary Information

Supplementary Material 1.

Supplementary Material 2.

## Data Availability

The full datasets produced and analyzed during this study are not publicly available in order to safeguard participant anonymity. However, they are available from the corresponding author upon reasonable request.

## Abbreviations

ANOVA: Analysis of variance
BMI: Body mass index
DM: Diabetes mellitus
DAS: Diabetes Attitude Scale
GPA: Grade point average
IM: Intramuscular
IRB: Institutional Review Board
IV: Intravenous
SC: Subcutaneous
SD: Standard deviation
