# Supplementary material for: Diabetes knowledge and attitudes among pharmacy students in Northern Cyprus: a cross-sectional study
Source: BMC Med Educ. 2026 Aug 12;26:1813. doi: 10.1186/s12909-026-10145-6 (PMC13746383; doi:10.1186/s12909-026-10145-6)
Supplement: Supplementary file 1 [file 12909_2026_10145_MOESM1_ESM.pdf]

SURVEY/ANKET

Assessment of Pharmacy Students' Knowledge and Attitude Regarding Diabetes Mellitus (Eczacılık öğrencilerinin Diyabetes Mellitus hakkındaki bilgi düzeyi ve tutumu değerlendirmesi)

Age / Yaş :

☐ Under 20/20 yaş altı ☐ 20–25 /20–25 yaş arası ☐ Above 25/ 25 yaş ve üstü

Gender/Cinsiyet:

☐ Male/Erkek ☐ Female/Kadın

Marital status/ Medeni hal:

☐ Single / Bekar ☐ In a relationship/ İlişkisi var ☐ Married/Evli

Academic Year/ Akademik Yıl:

☐ 4th ☐ 5th

GPA:

☐ < 2.0 ☐ 2.0–2.5 ☐ 2.5–3.0 ☐ > 3.0

BMI (kg/m2)/ Vücut Kitle İndeksi (VKİ) (kg/m2)

☐ < 18.5 ☐ 18.5–24.9 ☐ 25–29.9 ☐ ≥ 30

Mother's Education/ Annenizin Eğitim Düzeyi:

☐ Primary/İlkokul ☐ Secondary/Ortaokul ☐ High School/Lise ☐ University/Üniversite
☐ Graduate/Yüksek Lisans

Smoking/ Sigara Kullanımı

☐ Yes/Evet ☐ No/Hayır

Alcohol Use/Alkol Kullanımı:

☐ Yes/Evet ☐ No/Hayır

Do you exercise at least 6.5 h per week? Haftada en az 6.5 saat egzersiz yapar mısınız?

☐ Yes/Evet ☐ No/Hayır

Do you know someone with diabetes? Diyabet hastası tanıdığınız biri var mı?

☐ Yes/Evet ☐ No/Hayır

Yes/Evet **No/Hayır** **Don't know/Bilmiyorum**

General knowledge

	**Yes**	**No**	**Don't know**
**Is type 1 DM Insulin-dependent?**<br>Tip 1 DM İnsüline bağımlı mıdır?			
**Is type 2 DM Insulin resistant?**<br>Tip 2 DM İnsülin dirençli midir?			
**Only Type 1 DM presents with ketonuria?**<br>Sadece Tip 1 DM'de ketonüri görülür mü?			
**Are autoantibodies present in type 1 DM?**<br>Tip 1 DM'de otoantikorlar bulunur mu?			

RISK FACTORS/ RISK FAKTÖRLERI

**Age >45 years** / 1 Yaş >45 yıl			
**Obesity (BMI>30 kg/m2)/** Obezite (BMI>30 kg/m2)			
**Positive family history**/ Pozitif aile öyküsü			
**History of gestational diabetes**/ Gestasyonel diyabet öyküsü			
**Hypertension**/ Hipertansiyon			
**Carbohydrate-rich diet/**Karbonhidrat açısından zengin diyet			
**Lack of physical activity/** Yetersiz fiziksel aktivite			

SIGNS AND SYMPTOMS/ BELIRTILER VE SEMPTOMLAR

**Polyuria/** Poliüri			
**Thirst (polydipsia)/** Aşırı susama (polidipsi)			
**Dry mouth/** Ağız kuruluğu			
**Tiredness, fatigue, lethargy/** Yorgunluk, bitkinlik, letarji			
**Persistent hunger (Polyphagia)/** Aşırı yeme (Polifaji)			
**Delayed wound healing/** Yara iyileşmesinde gecikme			
**Frequent infections/** Sık enfeksiyon geçirme			

COMPLICATIONS/ KOMPLIKASYONLAR

	Yes	No	Don't know
**Retinopathy/** Retinopati			
**Cataract/** Katarakt			
**Renal failure/**Böbrek yetmezliği			
**Peripheral neuropathy/** Periferik nöropati			
**Ketoacidosis/** Ketoasidoz			
**Hypoglycemia/** Hipoglisemi			
**Hyperglycaemia/** Hiperglisemi			
**Limb gangrene/** Ekstremite gangreni			

Treatment and Diet/ Tedavi ve Diyet

Insulin is injected through: İnsülin şu yollarla enjekte edilir

☐ Intravenous/İntravenöz ☐ Intramuscular/İntramusküler ☐ Subcutaneous/Subkutan

A diabetic diet should include/ Diyabetik bir diyet şunları içermelidir

☐ 50% carbohydrate +35% fat +15% protein %50 karbonhidrat + %35 yağ + %15 protein

☐ 35% carbohydrate +50% fat +15% protein %35 karbonhidrat + %50 yağ + %15 protein

☐ 15% carbohydrate +35% fat +50% protein %15 karbonhidrat + %35 yağ + %50 protein

	**Strongly Agree** Kesinlikle Katılıyorum	**Agree** Katılıyorum	**Neutral** Nötr	**Disagree** Katılmıyorum	**Strongly Disagree** Kesinlikle Katılmıyorum
**Health care professionals who treat people with diabetes should be trained to communicate well with their patients.** Diyabetli kişilere bakım sağlayan sağlık profesyonelleri, hastalarıyla iyi iletişim kurabilmek için eğitilmelidir.					
**People who do not need to take insulin to treat their diabetes have a pretty mild disease.** Diyabetlerini tedavi etmek için insulin almaya ihtiyaç duymayan kişiler genellikle hafif bir hastalığa sahiptir.					
**There is not much use in trying to have good blood sugar control because the complications of diabetes will happen anyway.** İyi kan şekeri kontrolü sağlamaya çalışmanın pek bir faydası yoktur çünkü diyabetin komplikasyonları her durumda meydana gelebilir.					
**Diabetes affects almost every part of a diabetic person's life.**					

Diyabet, diyabetli bir kişinin yaşamının neredeyse her alanını etkiler.					
**The important decisions regarding daily diabetes care should be made by the person with diabetes.** Günlük diyabet bakımıyla ilgili önemli kararlar, diyabeti olan birey tarafından alınmalıdır.					
**Health care professionals should be taught how daily diabetes care affects patients.** Sağlık profesyonellerine günlük diyabet bakımının hastaları nasıl etkilediği öğretilmelidir					
**Older people with Type 2 diabetes do not usually get complications.** Tip 2 diyabeti olan yaşlı kişilerde genellikle komplikasyon görülmez.					
**Keeping the blood sugar close to normal can help to prevent the complications of diabetes.** Kan şekerini normale yakın tutmak diyabetin komplikasyonlarını önlemeye yardımcı olabilir.					
**Health care professionals should help patients make informed choices about their care plans.** Sağlık profesyonelleri, hastaların bakım planları hakkında bilinçli seçimler yapmalarına yardımcı olmalıdır.					
**It is important for the nurses and dietitians who teach people with diabetes to learn counselling skills.** Diyabetlilere eğitim veren hemşire ve diyetisyenlerin danışmanlık becerilerini öğrenmeleri önemlidir.					
**People whose diabetes is treated by just a diet do not have to worry about getting many long-term complications.** Diyabeti sadece diyetle tedavi edilen kişilerin uzun vadeli komplikasyonlar konusunda endişelenmelerine gerek yoktur.					
**Almost everyone with diabetes should do whatever it takes to keep their blood sugar close to normal.** Diyabetli hemen herkes kan şekerini normale yakın tutmak için ne gerekiyorsa yapmalıdır.					
**The emotional effects of diabetes are pretty small.** Diyabetin yarattığı duygusal etkiler oldukça azdır.					
**People with diabetes should have the final say in setting their blood glucose goals.** Diyabetli kişiler kan şekeri hedeflerini belirleme konusunda son sözü söylemelidir.					
**Blood sugar testing is not needed for people with Type 2 diabetes** Tip 2 diyabetli kişiler için kan şekeri testine gerek yoktur					
**Low blood sugar reactions make tight control too risky for most people.** Düşük kan şekeri reaksiyonları, çoğu kişi için sıkı kontrolü çok riskli hale getirir					
**Health care professionals should learn how to set goals with patients, not just tell them what to do.** Sağlık profesyonelleri, hastalara sadece ne yapacaklarını söylemek yerine, onlarla birlikte nasıl hedef belirleyeceklerini öğrenmelidir.					
**Diabetes is hard because you never get a break from it.** Diyabet zordur çünkü hiç ara vermeden devam eder.					

**The person with diabetes is the most important member of the diabetes care team.**<br>Diyabetli kişi, diyabet bakım ekibindeki en önemli üyedir.					
**Type 2 diabetes is a very serious disease.**<br>Tip 2 diyabet çok ciddi bir hastalıktır.					
**To do a good job, diabetes educators should learn a lot about being teachers**<br>İyi bir performans göstermek için, diyabet eğitimcilerinin öğretmenlik konusunda çok şey öğrenmeleri gerekir.					
**Having diabetes changes a person's outlook on life.**<br>Diyabetli olmak kişinin hayata bakış açısını değiştirir.					
**People who have Type 2 diabetes will probably not get much payoff from tight control of their blood sugars.**<br>Tip 2 diyabeti olan kişilerin kan şekerlerini sıkı bir şekilde kontrol etmelerinden muhtemelen çok fazla fayda görmeleri beklenmez.					
**People with diabetes should learn a lot about the disease so that they can be in charge of their own diabetes care.**<br>Diyabetli kişiler, kendi diyabet bakımlarından sorumlu olabilmeleri için hastalık hakkında çok şey öğrenmelidir.					
**Type 2 is as serious as Type 1 diabetes.**<br>Tip 2, Tip 1 diyabet kadar ciddidir.					
**Tight control is too much work.**<br>Sıkı kontrol, fazla iş gerektirir.					
**What the patient does has more effect on the outcome of diabetes care than anything a health professional does.**<br>Hastanın yaptıkları, diyabet bakımının sonuçları üzerinde sağlık profesyoneli tarafından yapılan her şeyden daha fazla etkiye sahiptir.					
**Tight control of blood sugar makes sense only for people with Type 1 diabetes.**<br>Kan şekerinin sıkı kontrolü, yalnızca Tip 1 diyabeti olan insanlar için mantıklıdır.					
**It is frustrating for people with diabetes to take care of their disease.**<br>Diyabetli kişiler için hastalıklarıyla ilgilenmek sinir bozucudur.					
**People with diabetes have a right to decide how hard they will work to control their blood sugar.**<br>Diyabeti olan insanların, kan şekerlerini kontrol etmek için ne kadar çaba harcayacaklarına kendileri karar verme hakkı vardır.					
**People who take diabetes pills should be as concerned about their blood sugar as people who take insulin.**<br>Diyabet ilaçları kullanan insanlar da, insülin kullanan insanlar kadar kan şekerleri konusunda dikkatli olmalılar.					
**People with diabetes have the right not to take good care of their diabetes.**<br>Diyabetli kişilerin diyabetlerine yeterince özen göstermeme hakları vardır.					
**Support from family and friends is important in dealing with diabetes.**<br>Aile ve arkadaşlardan gelen destek, diyabetle başa çıkmada önemlidir.					
