# Supplementary material for: Diabetes knowledge and attitudes among pharmacy students in Northern Cyprus: a cross-sectional study
Source: BMC Med Educ. 2026 Aug 12;26:1813. doi: 10.1186/s12909-026-10145-6 (PMC13746383; doi:10.1186/s12909-026-10145-6)
Supplement: Supplementary file 2 [file 12909_2026_10145_MOESM2_ESM.docx]

**Supplementary File 2**

**Table S1:** Item-total correlation coefficients for the knowledge questionnaire

| **Item** | **Question** | **Domain** | **Corrected Item–Total Correlation (r)** |
| --- | --- | --- | --- |
| Q1 | Are insulin-producing cells affected in T1DM? | General | 0.42 |
| Q2 | Can T1DM be prevented? | General | 0.58 |
| Q3 | Is T2DM characterised by insulin resistance? | General | 0.61 |
| Q4 | Does T1DM require lifelong insulin? | General | 0.39 |
| Q5 | Can diet prevent T2DM? | Risk Factors | 0.44 |
| Q6 | Is obesity a risk factor for T2DM? | Risk Factors | 0.52 |
| Q7 | Is physical inactivity a risk factor? | Risk Factors | 0.48 |
| Q8 | Is family history a risk factor? | Risk Factors | 0.55 |
| Q9 | Is ethnicity a risk factor? | Risk Factors | 0.41 |
| Q10 | Is polydipsia a symptom? | Signs/Symptoms | 0.63 |
| Q11 | Is polyuria a symptom? | Signs/Symptoms | 0.57 |
| Q12 | Is weight loss a symptom? | Signs/Symptoms | 0.59 |
| Q13 | Is blurred vision a symptom? | Signs/Symptoms | 0.46 |
| Q14 | Is fatigue a symptom? | Signs/Symptoms | 0.51 |
| Q15 | Can hypoglycaemia occur with insulin use? | Signs/Symptoms | 0.38 |
| Q16 | Is retinopathy a complication? | Complications | 0.62 |
| Q17 | Is nephropathy a complication? | Complications | 0.60 |
| Q18 | Is neuropathy a complication? | Complications | 0.58 |
| Q19 | Is cardiovascular disease a complication? | Complications | 0.64 |
| Q20 | Can DM cause foot ulcers? | Complications | 0.55 |
| Q21 | Do autoantibodies occur in T1DM? | General | 0.36 |
| Q22 | Is HbA1c used for monitoring? | General | 0.47 |
| Q23 | Are complex carbs preferred in DM diet? | Risk Factors | 0.43 |
| Q24 | Is 150 min/week exercise recommended? | Risk Factors | 0.39 |
| Q25 | Is BP control important in T2DM? | Complications | 0.52 |
| Q26 | Is aspirin used in DM prophylaxis? | Complications | 0.34 |

**Note:** All items demonstrated corrected item–total correlation coefficients greater than 0.30, indicating acceptable construct validity and supporting retention of all items in the final questionnaire.

**Table S2:** Internal Consistency Reliability of the Knowledge Questionnaire and DAS-3 Subscales

| **Instrument / Subscale** | **Items** | **Cronbach’s α** |
| --- | --- | --- |
| Knowledge Questionnaire (Overall) | 26 | 0.74 |
| General Knowledge | 6 | 0.61 |
| Risk Factors | 7 | 0.66 |
| Signs and Symptoms | 7 | 0.62 |
| Complications | 6 | 0.69 |
| Need for Special Training† | 5 | 0.51 |
| Patient Compliance | 6 | 0.66 |
| Seriousness of Type 2 Diabetes Mellitus | 5 | 0.62 |
| Value of Tight Glycaemic Control | 7 | 0.69 |
| Psychosocial Impact | 6 | 0.79 |
| Patient Autonomy | 4 | 0.79 |
| Team Care | 5 | 0.79 |

Notes: Cronbach’s α values were interpreted as follows: <0.60, poor; 0.60–0.69, questionable; 0.70–0.79, acceptable; ≥0.80, good. Interpretation thresholds were adapted from commonly accepted psychometric reliability criteria.

† The Need for Special Training subscale demonstrated relatively low internal consistency (Cronbach’s α = 0.51), which should be interpreted cautiously.

**Table S3:** Overall and Domain-Level Diabetes Knowledge Scores

| **Domain** | **Mean score** | **Correct responses (%)** |
| --- | --- | --- |
| General knowledge | 3.50/6 | 58.4% |
| Risk factors | 5.81/7 | 83.0% |
| Signs and symptoms | 5.78/7 | 82.5% |
| Complications | 4.30/6 | 71.7% |
| Overall knowledge | 19.20/26 | 73.8% |

**Table S4:** Item-Level Correct Response Rates and Equivalent Knowledge Scores

| **No.** | **Domain** | **Knowledge question** | **Correct answer** | **Total Correct**  **n/N (%)** | **Mean Score**  **n/26** |
| --- | --- | --- | --- | --- | --- |
| 1 | General knowledge | Is Type 1 DM insulin-dependent? | Yes | 218/265 (82.3%) | 21.39 |
| 2 | General knowledge | Is Type 2 DM insulin-resistant? | Yes | 204/265 (77.0%) | 20.02 |
| 3 | General knowledge | Does only Type 1 DM present with ketonuria? | No | 87/265 (32.8%) | 8.54 |
| 4 | General knowledge | Are autoantibodies present in Type 1 DM? | Yes | 155/265 (58.5%) | 15.21 |
| 5 | General knowledge | Insulin administration route | Subcutaneous (SC) | 182/265 (68.7%) | 17.86 |
| 6 | General knowledge | Diet recommendation | 50% carbohydrate + 35% fat + 15% protein | 83/265 (31.3%) | 8.14 |
| 7 | Risk factors | Age >45 years | Yes | 229/265 (86.4%) | 22.47 |
| 8 | Risk factors | Obesity (BMI >=30 kg/m²) | Yes | 230/265 (86.8%) | 22.57 |
| 9 | Risk factors | Positive family history | Yes | 233/265 (87.9%) | 22.86 |
| 10 | Risk factors | Gestational diabetes | Yes | 198/265 (74.7%) | 19.43 |
| 11 | Risk factors | Hypertension | Yes | 203/265 (76.6%) | 19.92 |
| 12 | Risk factors | Carbohydrate-rich diet | Yes | 211/265 (79.6%) | 20.70 |
| 13 | Risk factors | Lack of physical activity | Yes | 236/265 (89.1%) | 23.15 |
| 14 | Signs and symptoms | Polyuria | Yes | 217/265 (81.9%) | 21.29 |
| 15 | Signs and symptoms | Polydipsia | Yes | 238/265 (89.8%) | 23.35 |
| 16 | Signs and symptoms | Polyphagia | Yes | 213/265 (80.4%) | 20.90 |
| 17 | Signs and symptoms | Dry mouth | Yes | 224/265 (84.5%) | 21.98 |
| 18 | Signs and symptoms | Tiredness, fatigue, lethargy | Yes | 225/265 (84.9%) | 22.08 |
| 19 | Signs and symptoms | Delayed wound healing | Yes | 224/265 (84.5%) | 21.98 |
| 20 | Signs and symptoms | Frequent infections | Yes | 190/265 (71.7%) | 18.64 |
| 21 | Complications | Retinopathy | Yes | 200/265 (75.5%) | 19.62 |
| 22 | Complications | Nephropathy | Yes | 199/265 (75.1%) | 19.52 |
| 23 | Complications | Neuropathy | Yes | 189/265 (71.3%) | 18.54 |
| 24 | Complications | Ketoacidosis | Yes | 195/265 (73.6%) | 19.13 |
| 25 | Complications | Hypoglycemia | Yes | 181/265 (68.3%) | 17.76 |
| 26 | Complications | Limb gangrene | Yes | 176/265 (66.4%) | 17.27 |

**Note:** Correct responses were scored as 1 and incorrect or "Don't know" responses as 0. The equivalent score (/26) was calculated by multiplying the proportion of correct responses for each item by the maximum overall knowledge score (26 points). Domain and overall scores were derived from the mean proportion of correct responses across items within each domain.

**Table S5:** Attitudes of pharmacy students toward diabetes mellitus based on the Diabetes Attitude Scale (DAS)

| **Statement** | **4th year**  **(n =93), mean ± SD** | **5th year (n= 172), mean ± SD** | **p-value** |
| --- | --- | --- | --- |
| **In general, I believe that**: | | | |
| 1. ...health care professionals who treat people with diabetes should be trained to communicate well with their patients. | 4.82 ± 0.59 | 4.69 ± 0.72 | 0.152 |
| 2. ...people who do not need to take insulin to treat their diabetes have a pretty mild disease. **(negative)** | 2.35 ± 1.36 | 2.36 ± 1.16 | 0.972 |
| 3. ...there is not much use in trying to have good blood sugar control because the complications of diabetes will happen anyway. | 2.94 ± 1.47 | 3.15 ± 1.53 | 0.267 |
| 4. ...diabetes affects almost every part of a diabetic person’s life. | 4.30 ± 0.87 | 4.20 ± 0.97 | 0.390 |
| 5. ...the important decisions regarding daily diabetes care should be made by the person with diabetes. | 3.97 ± 1.05 | 3.87 ± 1.20 | 0.494 |
| 6. ...health care professionals should be taught how daily diabetes care affects patients’ lives. | 4.65 ± 0.72 | 4.60 ± 0.75 | 0.625 |
| 7. ...older people with Type 2* diabetes do not usually get complications. **(negative)** | 3.23 ± 1.34 | 3.26 ± 1.44 | 0.843 |
| 8. ...keeping the blood sugar close to normal can help to prevent the complications of diabetes. | 4.34 ± 0.88 | 4.44 ± 0.75 | 0.342 |
| 9. ...health care professionals should help patients make informed choices about their care plans. | 4.51 ± 0.87 | 4.55 ± 0.76 | 0.648 |
| 10. ...it is important for the nurses and dietitians who teach people with diabetes to learn counseling skills. | 4.51 ± 0.82 | 4.52 ± 0.76 | 0.859 |
| 11. ...people whose diabetes is treated by just a diet do not have to worry about getting many long-term complications. **(negative)** | 2.74 ± 1.32 | 2.76 ± 1.27 | 0.906 |
| 12. ...almost everyone with diabetes should do whatever it takes to keep their blood sugar close to normal. | 4.39 ± 0.83 | 4.32 ± 0.88 | 0.547 |
| 13. ...the emotional effects of diabetes are pretty small. **(negative)** | 3.14 ± 1.34 | 2.99 ± 1.37 | 0.406 |
| 14. ...people with diabetes should have the final say in setting their blood glucose goals. | 3.53 ± 1.29 | 3.38 ± 1.31 | 0.395 |
| 15. ...blood sugar testing is not needed for people with Type 2* diabetes. **(negative)** | 3.27 ± 1.43 | 3.30 ± 1.52 | 0.861 |
| 16. ...low blood sugar reactions make tight control too risky for most people. **(negative)** | 1.99 ± 0.94 | 2.16 ± 1.07 | 0.204 |
| 17. ...health care professionals should learn how to set goals with patients, not just tell them what to do. | 4.49 ± 0.82 | 4.47 ± 0.79 | 0.818 |
| 18. ...diabetes is hard because you never get a break from it. | 4.16 ± 0.94 | 4.10 ± 1.01 | 0.656 |
| 19. ...the person with diabetes is the most important member of the diabetes care team. | 4.28 ± 0.86 | 4.28 ± 1.05 | 0.997 |
| 20. ...to do a good job, diabetes educators should learn a lot about being teachers | 4.29 ± 0.94 | 4.31 ± 0.96 | 0.848 |
| 21. ...Type 2* diabetes is a very serious disease. | 4.18 ± 0.92 | 4.22 ± 0.93 | 0.749 |
| 22. ...having diabetes changes a person’s outlook on life. | 3.99 ± 1.06 | 4.06 ± 1.00 | 0.600 |
| 23. ...people who have Type 2* diabetes will probably not get much payoff from tight control of their blood sugars. **(negative)** | 2.94 ± 1.32 | 2.88 ± 1.31 | 0.760 |
| 24. ...people with diabetes should learn a lot about the disease so that they can be in charge of their own diabetes care. | 4.30 ± 0.93 | 4.32 ± 0.98 | 0.880 |
| 25. ...Type 2* is as serious as Type 1† diabetes. | 3.98 ± 1.08 | 4.12 ± 1.08 | 0.322 |
| 26. ...tight control is too much work. **(negative)** | 2.13 ± 0.99 | 2.19 ± 1.12 | 0.682 |
| 27. ...what the patient does has more effect on the outcome of diabetes care than anything a health professional does. | 4.04 ± 0.94 | 4.21 ± 0.96 | 0.178 |
| 28. ...tight control of blood sugar makes sense only for people with Type 1† diabetes. **(negative)** | 3.08 ± 1.31 | 3.05 ± 1.45 | 0.899 |
| 29. ...it is frustrating for people with diabetes to take care of their disease. | 3.63 ± 1.18 | 3.77 ± 1.07 | 0.330 |
| 30. ...people with diabetes have a right to decide how hard they will work to control their blood sugar. | 3.58 ± 1.14 | 3.67 ± 1.14 | 0.524 |
| 31. ...people who take diabetes pills should be as concerned about their blood sugar as people who take insulin. | 4.13 ± 1.01 | 4.28 ± 0.93 | 0.224 |
| 32. ...people with diabetes have the right not to take good care of their diabetes. | 2.94 ± 1.41 | 2.96 ± 1.50 | 0.900 |
| 33. ...support from family and friends is important in dealing with diabetes. | 4.46 ± 0.88 | 4.46 ± 0.86 | 0.978 |
